# Does size matter? Retrospective analysis of large gynecologic tumors

**DOI:** 10.4274/jtgga.2017.0022

**Published:** 2017-12-15

**Authors:** Tufan Öge, Emel Öztürk, Ömer T. Yalçın

**Affiliations:** 1 Department of Obstetrics and Gynecology, Eskişehir Osmangazi University School of Medicine, Eskişehir, Turkey; 2 Clinic of Obstetrics and Gynecology, Beyhekim State Hospital, Konya, Turkey

**Keywords:** Big gynecologic tumors, borderline tumors, leiomyoma, ovarian cysts

## Abstract

**Objective:**

To evaluate the characteristics of patients who underwent surgery due to the presence of a large pelvic-abdominal mass over a 5-year period in a university clinic.

**Material and Methods:**

Among 3476 gynecologic operations, intraoperative findings were evaluated retrospectively. Uterine and/or adnexal masses smaller than 20 cm were excluded to refine “large” tumors and 74 patients with large tumors were enrolled in the study group. Demographic characteristics, intraoperative findings, and results of histopathologic examinations were recorded. Moreover, preoperative and intraoperative findings were compared among tumors with adnexal origin according to their final histopathologic results.

**Results:**

The mean age of the patients was 46 years. The most common symptom was abdominal pain, as recorded in 38 (51.4%) patients. Among all patients, 31 (41.9%) had coexisting illness and 13 (17.6%) had a history of surgery. The mean tumor diameter was 25.9±8.6 cm (20-60) and 60 (78.9%) tumors were of adnexal origin. The ratios of malignancy for large adnexal and uterine tumors were 34.4% and 12.5%, respectively. When the large adnexal tumors were re-evaluated, the mean cancer antigen (CA) 125 level was significantly higher, and ascites was more frequently detected in malignant tumors (p<0.01) then in benign and borderline tumors.

**Conclusion:**

Benign and borderline tumors are more common among large abdominopelvic masses, although the presence of ascites and elevated CA 125 may present malignancy in large gynecologic tumors. Further studies with larger sample sizes are needed to define the characteristics of large tumors and their malignant potentials.

## INTRODUCTION

Managing pelvic masses, which may result from benign or malignant conditions of gynecologic and non-gynecologic diseases, is routine daily practice for outpatient clinic gynecologists. However, due to its rarity, large-sized pelvic tumors that reach the upper abdomen may sometimes be confusing for physicians (1,2). The size of the adnexal mass is one of the important factors in making decisions for the clinical management, and therefore some indexes have to be taken into account (3,4,5,6,7). Although “giant”, “huge,” and “large” tumor descriptions are not very clear, and the cut-off sizes of these explanations are inconclusive, gynecologists and oncologists can diagnose large pelvic-abdominal masses that require treatment.

To our knowledge, although the English literature consists of case reports including large gynecologic tumors of ovarian, tubal, and uterine origin, there is lack of data regarding a series of large tumors (8,9,10). From this point of view, we aimed to evaluate the characteristics of patients who were diagnosed as having and underwent surgery for large pelvic-abdominal masses over a 5-year period in a university clinic.

## MATERIAL AND METHODS

After obtaining approval from the local ethics committee, the surgical and pathologic reports of patients who underwent surgical procedures due to suspected adnexal masses between 2011 and 2016 were retrospectively reviewed in our university clinic.

**Figure 1:**
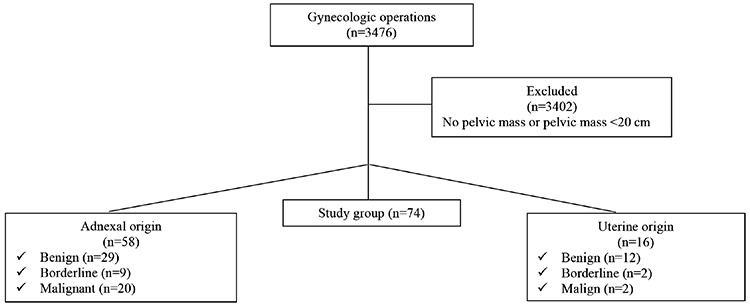
Flow chart of the patients

All patients underwent detailed pelvic examination, ultrasonographic evaluation and computerized tomography or magnetic resonance imaging (MRI) if needed. Thereafter, all patients were evaluated by the tumor board and surgery indications were approved. After preoperative medical evaluations, all patients underwent laparotomy through a midline incision. Adnexal masses with suspected malignancies were sent for frozen section. According to frozen section histopathologic evaluations, patients with malignant masses underwent debulking surgery, and patients with benign disease underwent conservative surgery or total hysterectomy and salpingo-oophorectomy. Among 3476 gynecologic operations, intraoperative findings were evaluated and uterine and/or adnexal masses smaller than 20 cm were excluded to refine “large” tumors, and 74 patients with large tumors were enrolled in the study group. A flow chart of the patients is shown in Figure 1.

Demographic characteristics including age, gravidity, parity, medical history, symptoms, physical and pelvic examination, ultrasonographic evaluations, cancer antigen (CA) 125 levels, intraoperative findings, and results of the histopathologic examinations of the patients were recorded. In addition, preoperative and intraoperative findings were compared among tumors with adnexal origin according to their final histopathologic results.

Statistical analysis of the collected data was performed using IBM Statistical Package for Social Sciences (SPSS) 23.0 software. The normality of distribution was checked initially using Shapiro-Wilk’s test and parametric or non-parametric tests were applied to data with normal or non-normal distribution, respectively. One-way analysis of variance (ANOVA) with Tukey’s honest significant difference post hoc test and Kruskal-Wallis (ANOVA on Ranks) tests with Dunn’s post hoc tests were applied to determine the differences among benign, borderline, and malignant adnexal masses. Chi-square tests were applied for categorical variables. The results are expressed as mean ± standard deviation and median (interquartile range Q1 and Q3); p values <0.05 were considered statistically significant.

## RESULTS

The mean age of the patients was 47 years. Of the patients, 54.1% (n=40) were premenopausal and 45.9% (n=40) were postmenopausal. Two of the premenopausal patients were adolescents. The most common symptom was abdominal pain, which was recorded in 38 (51.4%) patients. Among all patients, 31 (41.9%) had coexisting illness and 13 (17.6%) had a history of surgery. The patients’ demographic characteristics, preoperative CA 125, and hemoglobin levels are summarized in Table 1. According to the operation findings, the mean tumor diameter was 25.9±8.6 cm (20-60) and 60 (78.9%) of the tumors were of adnexal origin. During the operations, adhesiolysis was performed in 30 (40.5%) operations, 4 small bowel lacerations, 1 bladder, and 1 sigmoid perforation occurred, all were repaired in the same session. Exploration findings during laparotomy are summarized in Table 2. Among 74 operations, 58 of 60 adnexal masses were sent for frozen section analysis. None of the large tumors of uterine origin (n=14) were evaluated using frozen section analysis. Frozen section examination revealed benign, borderline, and malignant tumors in 29 (50%), 9 (15.5%), and 20 (34.4%) patients with adnexal masses, respectively. Histopathologic examination revealed leiomyosarcoma in 2 (12.5%) of 16 patients with gynecologic tumors of uterine origin. When patients were classified according to menopausal status, final histopathologic examinations revealed malignancy in 13 of the 34 (38.2%) menopausal patients, and 9 of the 40 (22.5%) pre-menopausal patients. Moreover, 2 of the pre-menopausal patients were adolescents and their evaluations were regarded as benign disease. The ratios of malignancy for large adnexal and uterine tumors were 34.4% and 12.5%, respectively. Detailed final histopathologic examination distributions are shown in Table 3.

When the adnexal big tumors were re-evaluated, the mean CA 125 level was significantly higher, and ascites was more frequently detected in malignant tumors (p<0.01) than in benign and borderline tumors. The characteristics of the large adnexal tumors according to final histopathologic results are summarized in Table 4.

## DISCUSSION

In the present study, we aimed to evaluate and share our experience of large abdominopelvic tumors that underwent surgery in a university gynecology clinic. Of the tumors with adnexal and uterine origin, 34.4% and 12.5% were found to be malignant, and 15.5% and 12% of tumors were diagnosed as borderline ovarian and uterine smooth muscle tumors of uncertain malignant potential, respectively. The mean size and weight of tumor were not statistically different between benign, borderline, and malignant large tumors; however, CA 125 was found to be elevated, and the presence of ascites was significantly detected in large malignant tumors.

One of the limitations of this study is its retrospective design. All operation notes were evaluated but there may still have been patients who did not undergo surgery or were lost before the operation. Moreover, our cut-off limit may be questionable; however, to our knowledge, there is no consensus for the size of tumors to call them large, huge, or giant. Therefore, after searching the literature and our patients’ charts, we set out cut-off level for "large" tumors at 20 cm, and evaluated the operative characteristics of the patients in order to share our experience of large tumors. Also, we included all uterine and adnexal tumors in our study because we aimed to evaluate all large-sized tumors. Before surgery, all patients underwent ultrasonography and there was no question as to whether the origin was uterus or adnexa. Another limitation of this study is the sample size, even though large tumors are not very common. Our sample size was very small for large tumors with uterine origin; therefore, we only compared the operative characteristics of patients with large adnexal tumors.

During gynecology and gynecologic oncology practice, physicians usually diagnose adnexal masses and the most important issue it to exclude malignancy during the management. Ultrasonographic evaluation, menopausal status, and tumor markers such as CA 125 and HE4 are important predictors for malignancy (4,11). Moreover, the presence of a multi­locular cystic lesions, solid areas, bilateral lesions, ascites, and intra-abdominal metastases are also known to be important parameters during ultrasonographic evaluations. These features are known to be important morphologic features of adnexal masses. In addition, unilocular tumors, smooth multilocular tumors and no intra-tumoral blood flow in color or power Doppler are simple rules to predict benign disease, whereas irregular solid tumor, ascites, at least 4 papillary projections, and strong intra-tumoral blood flow in color or power Doppler may predict malignancy (12). Efforts are ongoing to standardize ultrasonographic evaluations for optimal patient management (13). For larger tumors, computed tomography (CT) or MRI may be required to determine the origin of the tumor. In the present study, 64 (86.4%) patients were evaluated using CT or MRI for the differential diagnose. MRI was preferred especially for suspected masses of uterine origin in 10 (13.5%) patients, whereas 54 (72.9%) patients were evaluated using CT to discriminate the origin of the tumor, possible metastasis, and predict optimal cytoreduction.

In addition, some scoring systems such as the Rajavithi-Ovarian Cancer Predictive Score [risk of ovarian malignancy algorithm and risk of malignancy index (RMI)] have been introduced to discriminate benign and malignant cases (3,4,6). RMI is the one of the most common methods using the knowledge of menopausal status (M), ultrasound findings (U), and the serum CA 125 level, and is calculated as M × U × CA 125; (a total ultrasound score of 0 yields U=0, a score of 1 yields U=1, and a score of 2 yields U=3. Premenopausal status yields M=1, and post-M yields M=3. The serum level of CA 125 is applied directly to the calculation (6,7). RMI is then developed as RMI 1, RMI 2, and RMI 3 using the same formula, but scoring differently, and RMI 4 where size (S) is taken into account in a formula as S × M × U × CA 125. RMI 4 takes tumor size <7 cm as S=0, and ≥7 cm as S=2 in the formula, and was introduced to be more reliable than RMI 1, RMI 2, and RMI 3. However, the diagnostic accuracy is still inconclusive in large tumors and new studies are needed to evaluate this issue.

Although there are some protocols or indexes for the management of adnexal masses, frozen section analysis is usually mandatory for large tumors. Large tumors typically require a midline incision reaching the upper abdomen, and need extra care during operations, but the role of minimally invasive surgery cannot be ignored. In some studies, laparoscopy was suggested a feasible and safe treatment for women with large ovarian cysts with proper patient selection (14,15,16). However, surgeons should carefully consider the potential risk of malignancy in such patients, and surgeon experience may still be a limitation with large tumors.

Mucinous tumors are more likely present in large masses averaging 16 to 30 cm in diameter (17,18). A retrospective study evaluating mucinous borderline tumors reported the median tumor size as 20 com (range, 4-40 cm) (19). In the present study, of the 9 borderline tumors, 7 (77%) were mucinous, the mean diameter was 26 cm. Although mucinous ovarian cancer is an uncommon subtype of malignant ovarian tumors and accounts for approximately 5% to 10% of ovarian carcinomas, we found that the most frequent histologic type was mucinous adenocarcinoma. This finding may be due to our selection criteria, only choosing masses 20 cm and above.

To conclude, physicians should be aware of the malignancy potential and plan the optimal surgical team and procedure because large gynecologic tumors require surgical treatment. Benign and borderline tumors are more common among large abdominopelvic masses although the presence of ascites and elevated CA 125 may present malignancy in large gynecologic tumors. Further studies with larger sample sizes are needed to define the characteristics of large tumors and their malignant potentials.

## Figures and Tables

**Table 1:**
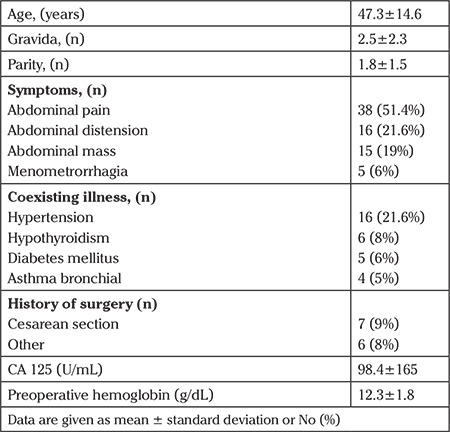
Patient characteristics

**Table 2:**
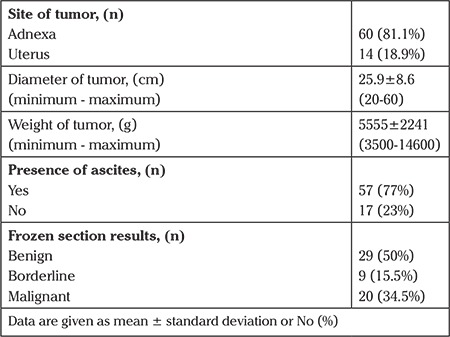
Exploration findings during laparotomy

**Table 3:**
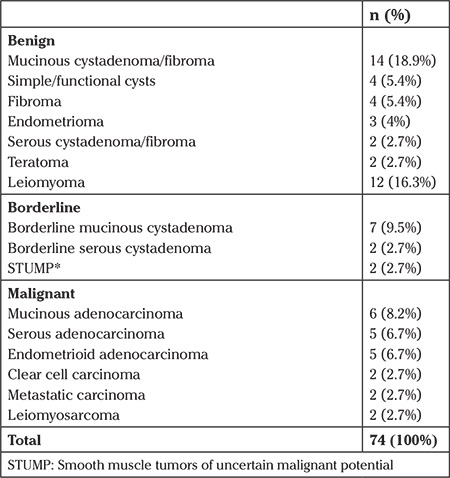
Final histopathologic results

**Table 4:**
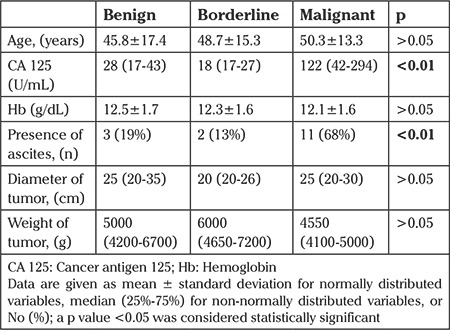
Characteristics of big tumors with adnexal origin according to final histopathologic results
